# In Vitro Anti-Inflammatory Activity of Essential Oil and β-Bisabolol Derived from Cotton Gin Trash

**DOI:** 10.3390/molecules27020526

**Published:** 2022-01-14

**Authors:** Mary A. Egbuta, Shane McIntosh, Daniel L. E. Waters, Tony Vancov, Lei Liu

**Affiliations:** 1 Southern Cross Plant Science, Faculty of Science and Engineering, Southern Cross University, Lismore, NSW 2480, Australia; Mary.Egbuta@scu.edu.au (M.A.E.); Shane.McIntosh@scu.edu.au (S.M.); daniel.waters@scu.edu.au (D.L.E.W.); 2 Elizabeth Macarthur Agricultural Institute, NSW Department of Planning, Industry & Environment, DPI Agriculture, Woodbridge Rd, Menangle, NSW 2568, Australia; tony.vancov@dpi.nsw.gov.au

**Keywords:** β-bisabolol, cotton gin trash, anti-inflammatory, cytokines, cells, nitric oxide

## Abstract

Natural α-bisabolol has been widely used in cosmetics and is sourced mainly from the stems of Candeia trees that have become endangered due to over exploitation. The in vitro anti-inflammatory activity of cotton gin trash (CGT) essential oil and the major terpenoid (β-bisabolol) purified from the oil were investigated against lipopolysaccharide (LPS)-stimulated RAW264.7 macrophages as well as the 3t3 and HS27 fibroblast cell lines. Nitric oxide (NO), prostaglandin E_2_ (PGE_2_), tumor necrosis factor-alpha (TNF-α), interleukin 6 (IL-6), and interleukin 8 (IL-8) were measured using Greiss reagent, enzyme-linked immunosorbent assay (ELISA), and cytokine bead array (CBA)-flow cytometry. Non-toxic concentrations of CGT oil and β-bisabolol (1.6–50.0 µg/mL) significantly inhibited the production of the inflammatory mediators in a dose-dependent manner. Maximal inhibition by β-bisabolol was 55.5% for NO, 62.3% for PGE_2_, and 45.3% for TNF-α production in RAW cells. β-Bisabolol induced a level of inhibition similar to an equal concentration of α-bisabolol (50.0 µg/mL), a known anti-inflammatory agent. These results suggest β-bisabolol exerts similar in vitro effects to known topical anti-inflammatory agents and could therefore be exploited for cosmetic and therapeutic uses. This is the first study to report the in vitro anti-inflammatory activity of β-bisabolol in CGT essential oil.

## 1. Introduction

Inflammation is a primary part of the complex reaction of the human body to the presence of harmful stimuli such as pathogens or injury [1,2]. During the process of inflammation, mediators such as nitric oxide (NO), prostaglandin E_2_ (PGE_2_), and cytokines are produced by activated cells [3]. Inflammation mediators induce a cascade of reactions that involve stimulating other cells to respond to the offending pathogen [4,5]. Normal inflammatory reactions, also referred to as acute inflammation, are terminated once the function of inflammation has been accomplished, with downregulation of pro-inflammatory mediators and upregulation of anti-inflammatory mediators [1,6]. However, uncontrolled inflammation, also known as chronic inflammation, can lead to cellular and tissue damage, causing chronic diseases [7,8]. Regulating the production of mediators plays an important role in inflammatory responses and disease management such that compounds with anti-inflammatory properties are constantly investigated for this purpose.

Inflammation mediators such as NO, PGE_2_, interleukin 6 (IL-6), tumor necrosis factor-alpha (TNF-α), and interleukin 8 (IL-8) are some examples of cell signaling molecules that promote prolonged inflammation [9,10,11], and if uncontrolled can result in chronic inflammation [12]. Initiated by the enzyme inducible nitric oxide synthase (iNOS), nitric oxide is produced at different stages of inflammation, either at the onset or mid-way through the reaction [5,13] as well as stimulating production of other inflammation mediators such as TNF-α and interleukin 1-α (IL1-α) [14]. PGE_2_, one of four known prostaglandins, is a lipid inflammatory mediator produced as a result of activity of the cyclooxygenases (COX1 and COX2) and prostaglandin synthases [15,16]. They are active contributors to inflammation, playing a key role in the generation of the process [16,17]. PGE_2_ is the most abundant prostaglandin (PG) produced in the body and its biosynthesis increases in inflamed tissues, thereby initiating the production of other pro-inflammatory mediators such as interleukin 17 cytokine (IL-17), which activates neutrophils and monocytes to sites of infection [17,18]. The pro-inflammatory cytokines, TNF-α and IL-6, are also key players in the process of inflammation. Interleukin 6 acts as a pro-inflammation mediator [19,20] initiating the acute phase of inflammation [21,22], and TNF-α is another important pro-inflammation mediator that is actively involved in innate and adaptive immunity [23,24].

Production of these mediators are specific to certain cells including macrophages [25] and fibroblasts [26,27,28]. Macrophages, differentiated monocytes, and highly versatile large white blood cells contribute to inflammation at all stages of the reaction [29,30]. Macrophages help to initiate and terminate inflammation by enabling the elimination of offending factors and stimulating subsequent tissue repair [31,32]. This is because macrophages possess two phenotypes, M1 and M2, which have different functions [25]. M1 macrophages are pro-inflammatory in nature [30,31], secreting cytokines such as TNF-α, IL-6, and interleukin 12 (IL-12), whereas, M2 macrophages are anti-inflammatory in nature, producing interleukin 10 (IL-10) and transforming growth factor β (TGF-β) [32,33,34].

Fibroblasts are also active in the process of inflammation. In addition to their mechanical and structural roles in tissues, they also promote inflammation by producing cytokines (e.g., IL-1β and IL-6) and chemokines (e.g., IL-8) when stimulated [27,35,36], activating and migrating macrophages and neutrophils to the points of infection [26,37]. A distortion to the normal activity of these cells in the inflammation process results in prolonged secretion of pro-inflammatory cytokines, which eventually leads to disease [30]. Anti-inflammatory agents, both natural and synthetic, have been used to assist the body system in controlling the excessive activity of these inflammation mediators [38,39].

Plant-derived natural compounds are constantly being investigated for anti-inflammatory properties [40,41], particularly because natural products are starting points for the development of therapeutic agents and synthetic medicinal chemistry [1]. Terpenoids are constituents of essential oils derived from plants and are an example of such medically important natural products [42,43,44].

Plant derived essential oils are exploited for their anti-inflammatory properties and have been shown to be effective using different cell lines [45,46]. The terpenoids found in essential oils including monoterpenoids and sesquiterpenoids have been reported to inhibit the production of inflammatory mediators such as NO, PGE_2_, TNF-α, IL-6, and IL-8 in in vitro anti-inflammatory studies [47]. Inhibition of these inflammation mediators by the volatile chemicals is due to the inhibition of enzymes that initiate the production of the mediators [2]. An example of inflammatory enzyme inhibition by terpenoids include α-humulene and β-caryophyllene inhibition of COX-2 and iNOS production by carrageenan-treated rat paw [48]. Another example is the sesquiterpenoid, α-bisabolol, which was reported by Maurya et al. [49] to inhibit the production of iNOS and COX-2 during LPS-induced inflammation of RAW 264.7 cells.

In our previous publication [50], essential oil extracted from cotton gin trash samples were analyzed for their chemical composition following different extraction procedures. Compounds in the extracted oil included terpenoids such as α-humulene and β-caryophyllene, known in the literature to be biologically active, with β-bisabolol being the most abundant. Although some of the terpenoids identified in CGT oil have anti-inflammatory activities, [51] and β-bisabolol’s isomer, α-bisabolol, has been reported to have bioactive properties such as anti-cancer [52,53] and anti-inflammation [54,55], knowledge on the biological activity of β-bisabolol is limited. This study investigated the anti-inflammatory activity of CGT oil and its major terpenoid, β-bisabolol on stimulated fibroblasts and macrophages. The anti-inflammatory activity of β-bisabolol was compared against that of its isomer α-bisabolol.

## 2. Results

### 2.1. Composition of CGT Oil

The extraction yield of the essential oil was about 0.1% of CGT (m/m), similar to our previously reported yield. [50] The dominant non-fatty acid compounds in the essential oil extracted from pesticide-free CGT were terpenoids. Terpenoid composition of CGT oil included monoterpenoids such as α-pinene (1.3%), myrcene (1.1%), and β-(E)-ocimene (3.9%) and sesquiterpenoids such as β-caryophyllene (6.8%), α-humulene (4.1%), caryophyllene oxide (4.7%), β-bisabolol (23.5%), and γ-bisabolene (8.7%) (Table 1). The sesquiterpenoids were the most abundant in the oil, making up 62.0% of the total peak areas calculated by GC-MS analysis, and β-bisabolol was the most abundant volatile compound, making up approximately a quarter of the volatiles in CGT oil analyzed by GC-MS (Supplementary Table S1).

Percentage composition of terpenoids calculated from the total area under peaks of all volatiles identified in the CGT oil extract (Supplementary Table S1).

### 2.2. Effect of CGT Oil and Isolated Compound (β-Bisabolol) on Cell Viability

**Figure 1:**
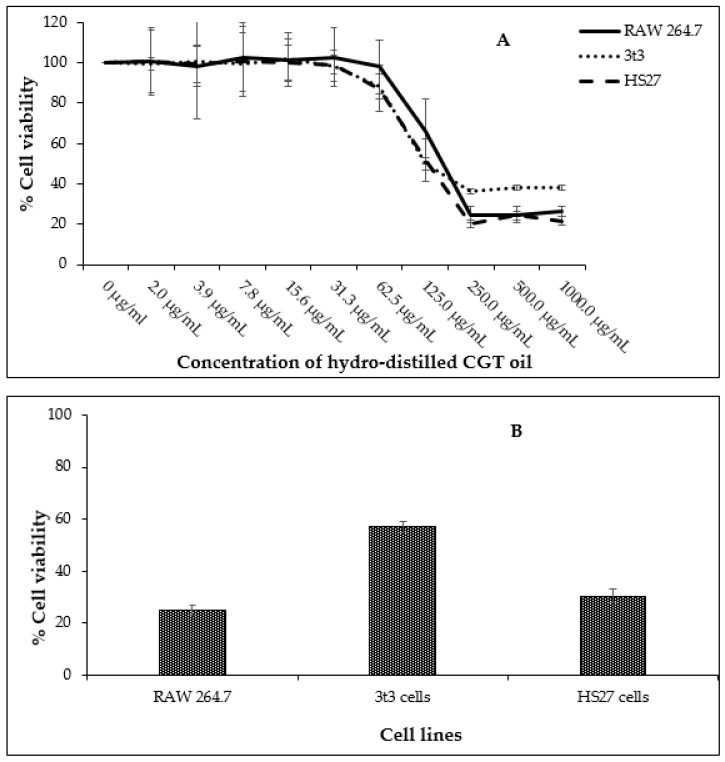
Cell viability of RAW 264.7, HS27, and 3t3 cells (*n* = 3) exposed to different concentrations of CGT oil (1:2 dilution) for 24 h (**A**) and cell viability recorded against the positive control chlorambucil (**B**, at 1 mg/mL). Percentage cell viability is represented as mean ± SD.

Fibroblasts (HS27 and 3t3) and leukemic macrophages (RAW 264.7) were exposed to concentrations of CGT oil ranging from 2.0 µg/mL to 1000.0 µg/mL to determine a non-toxic concentration range of the oil for further anti-inflammatory assays. The highest concentration of CGT oil (1000.0 µg/mL) induced a similar toxic effect as the positive control chlorambucil (1 mg/mL) (Figure 1). The half maximal inhibitory concentration (IC_50_) of CGT oil was calculated to be 129.4 µg/mL for RAW 264.7, 90.2 µg/mL for 3t3, and 107.2 µg/mL for HS27 cells.

**Figure 2:**
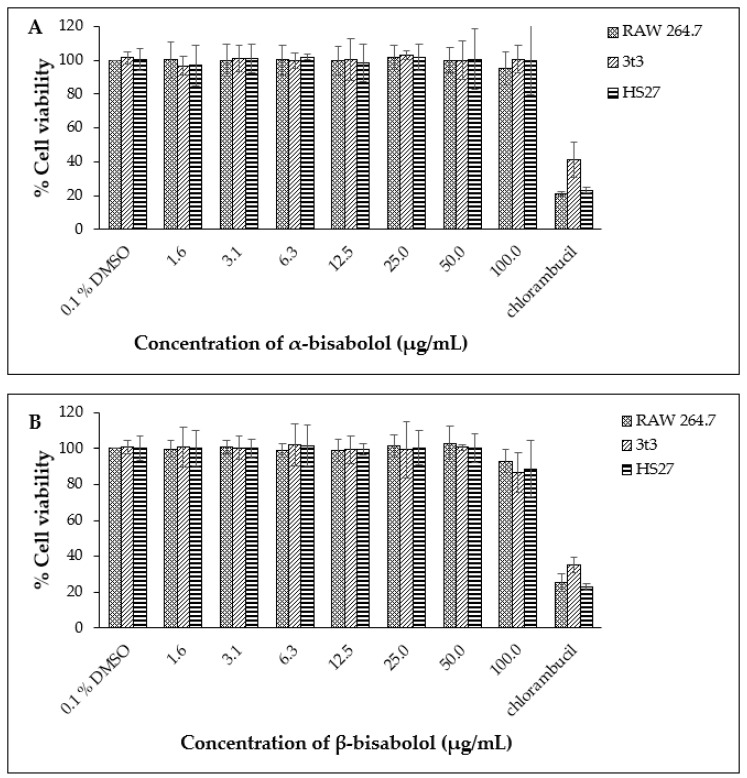
Cell viability observed for RAW 264.7, 3t3 and HS27 cells (*n* = 3) exposed to α-bisabolol (**A**) and β-bisabolol (**B**) concentrations for 24 h against the positive control chlorambucil (1 mg/mL). Percentage cell viability is represented as mean ±SD.

Based on these data, the non-toxic range was deemed to be from 1.6 µg/mL to 50.0 µg/mL. The non-toxic concentration of β-bisabolol and α-bisabolol was selected following the anti-inflammatory assay of Maurya et al. [49], who reported that concentrations as high as 100 µg/mL were non-toxic to peritoneal macrophages. To confirm these reports, cell viability assays were performed using a similar concentration range (1.6 µg/mL to 100.0 µg/mL). Concentrations of α- and β-bisabolol as high as 50 µg/mL (Figure 2) did not affect cell viability whereas 100 µg/mL β-bisabolol was slightly toxic to all three cell lines. 

### 2.3. Nitric Oxide Inhibition of CGT Oil and β-Bisabolol on RAW 264.7 Cells

**Figure 3:**
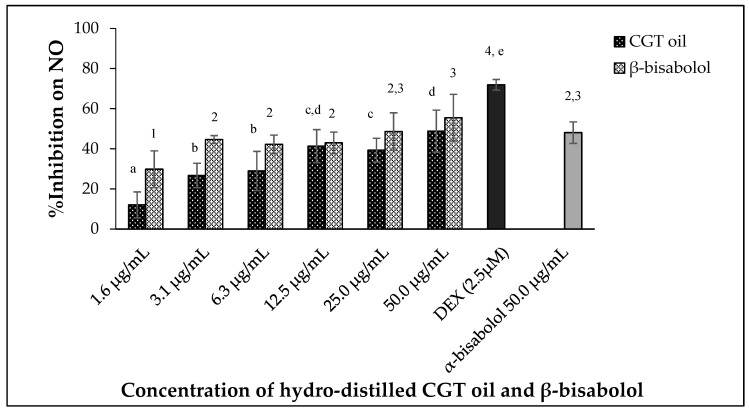
Effect of CGT oil and β-bisabolol on NO production in RAW 264.7 cells. RAW 264.7 cells were exposed to test extracts or compounds for 1 h and then induced with 100 ng/mL lipopolysaccharide (LPS) for NO production (*n* = 3). Values are expressed as mean ± SD. Duncan’s multiple range test between average NO inhibition across the different concentrations indicates that dissimilar letters/numerals denote a significant difference (*p* ≤ 0.05).

CGT oil and β-bisabolol significantly regulated the production of nitric oxide by the macrophages in a dose-dependent manner (Figure 3) after 24 h of exposure (*p* < 0.05). The effectiveness of CGT oil to regulate the production of NO was such that there was an observed reduction of 12.0% at the lowest concentration of the oil (1.6 µg/mL). At the maximum concentration of CGT oil (50.0 µg/mL), NO was inhibited by 48.8% compared to 71.9% NO inhibition induced by 2.5 µM of the anti-inflammatory drug, dexamethasone (DEX). 

Nitric oxide production was reduced by 29.8% by the lowest concentration of β-bisabolol (1.6 µg/mL) and inhibition of NO remained unchanged when RAW 264.7 macrophages were exposed to 3.1 µg/mL and 12.5 µg/mL β-bisabolol (Figure 3). The highest concentration of β-bisabolol (50 µg/mL) reduced NO production by 55.5%, which was more than the effect of the same concentration of CGT oil by 6.7%. When comparing the activity of 50.0 µg/mL β-bisabolol against α-bisabolol of similar concentration (50.0 µg/mL) (Figure 3), β-bisabolol was a more effective inhibitor inhibiting nitrate production by 55.5%, whereas α-bisabolol at 50 µg/mL inhibited NO production by only 48.1%.

### 2.4. Inhibition of PGE_2_ Production by CGT Oil and β-Bisabolol

**Figure 4:**
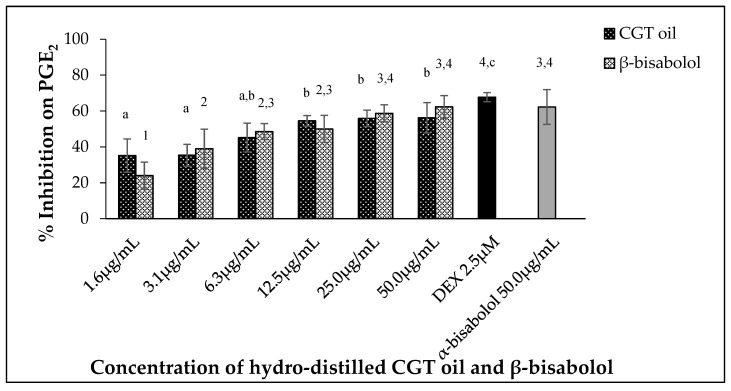
Effect of CGT oil and β-bisabolol dilutions on PGE_2_ production in RAW 264.7 cells. RAW 264.7 cells were exposed to test extracts or compounds for 1 h and then induced with 100 ng/mL lipopolysaccharide (LPS) for PGE_2_ production (*n* = 3). Values are expressed as mean ± SD. Duncan’s multiple range test between average NO production across the different concentrations indicates that dissimilar letters/numerals denote a significant difference (*p* ≤ 0.05).

Leukemic macrophages (RAW 264.7) used in this study were able to release PGE_2_ when stimulated by 100 ng/mL LPS. Oil extracted from CGT reduced the production of PGE_2_ in a dose-dependent manner (*p* < 0.05) (Figure 4). The same concentration-dependent response was also observed for β-bisabolol (Figure 4) as 1.6 µg/mL reduced PGE_2_ production by 24.0% and 50.0 µg/mL by 62.3%.

### 2.5. CGT Oil and β-Bisabolol Inhibit Pro-Inflammatory Cytokine Production

#### 2.5.1. Interleukin 6 (IL-6) Inhibition

**Figure 5:**
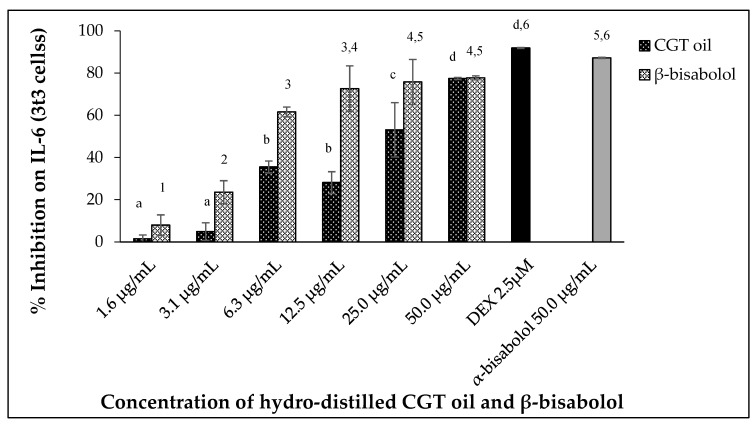
Effect of CGT oil and β-bisabolol dilutions on IL-6 production in 3t3 cells. Cells were exposed to test extracts or compounds for 1 h and then induced with 100 ng/mL lipopolysaccharide (LPS) for IL-6 production (*n* = 3). Values are expressed as mean ± SD. Duncan’s multiple range test between average IL-6 production across the different concentrations indicates that dissimilar letters/numerals denote a significant difference (*p* ≤ 0.05).

Uncontrolled secretion of Interleukin 6 (IL-6) can result in chronic inflammation and disease. In this study, mouse 3t3 cells produced IL-6 when stimulated with 100 ng/mL LPS after 24 h. Exposure of the cells (3t3) to increasing concentrations of CGT oil revealed an increase in IL-6 inhibition (Figure 5). Exposure to cotton gin trash oil reduced IL-6 secretion by 1.5% at the lowest concentration and 77.4% at the highest (Figure 5). The response of cells exposed to increasing concentrations of β-bisabolol also showed a dose-dependent reduction in the production of IL-6 by the fibroblasts, inhibiting secretion of IL-6 from 7.9% to 77.7% (Figure 5).

The effect of CGT oil and β-bisabolol on 3t3 IL-6 production was significant (*p* < 0.05), with an EC_50_ of 27.2 µg/mL and 4.3 µg/mL, respectively. Comparing the response of LPS-stimulated cells to 50.0 µg/mL of either β-bisabolol or α-bisabolol indicated there was a significant difference between the two responses with the presence of α-bisabolol resulting in greater inhibition of IL-6 (87.2%), 9.5% more than its isomer β-bisabolol, which inhibited IL-6 production by 77.7%. Nevertheless, inhibition of IL-6 production by β-bisabolol is indicative of its anti-inflammatory activity. 

**Figure 6:**
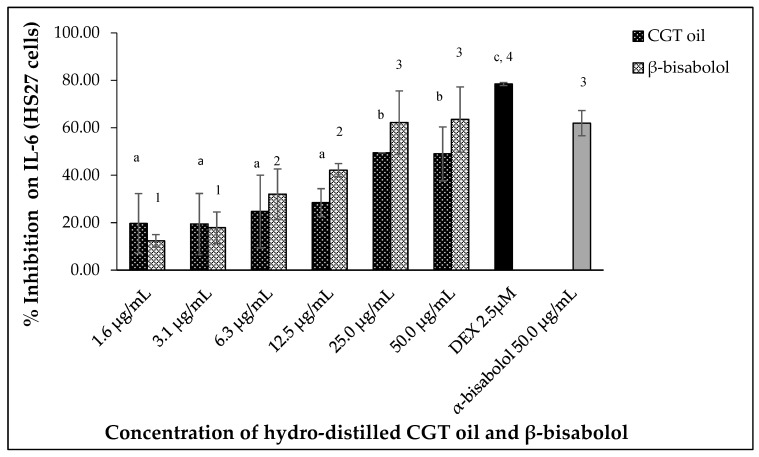
Effect of CGT oil and β-bisabolol dilutions on IL-6 production in HS27 cells. HS27 cells were exposed to test extracts or compounds for 1 h and then induced with 100 ng/mL lipopolysaccharide (LPS) for IL-6 production (*n* = 3). Values are expressed as mean ± SD. Duncan’s multiple range test between average IL-6 production across the different concentrations indicates that dissimilar letters/numerals denote a significant difference (*p* ≤ 0.05).

Lipopolysaccharide (LPS)-stimulated human fibroblasts (HS27) also produced IL-6 and inhibition of the cytokine was observed after 24 h exposure to CGT oil and β-bisabolol. Hydro-distilled CGT oil concentrations inhibited IL-6 production by up to 49.5% at the highest concentration of 50.0 µg/mL (Figure 6). β-Bisabolol concentrations also inhibited the production of the same cytokine of up to 63.5% at the highest concentration of 50.0 µg/mL (Figure 6). At the lowest concentration of 1.6 µg/mL for both CGT oil and β-bisabolol, IL-6 inhibition was 19.6% and 12.4%, respectively. EC_50_ values for CGT oil and β-bisabolol in HS27 cells were 15.0 µg/mL and 8.8 µg/mL, respectively. Production of IL-6 by HS27 cells was inhibited by 50.0 µg/mL of β-bisabolol at a similar level to what was observed for α-bisabolol. 

#### 2.5.2. Interleukin 8 (IL-8) Inhibition

**Figure 7:**
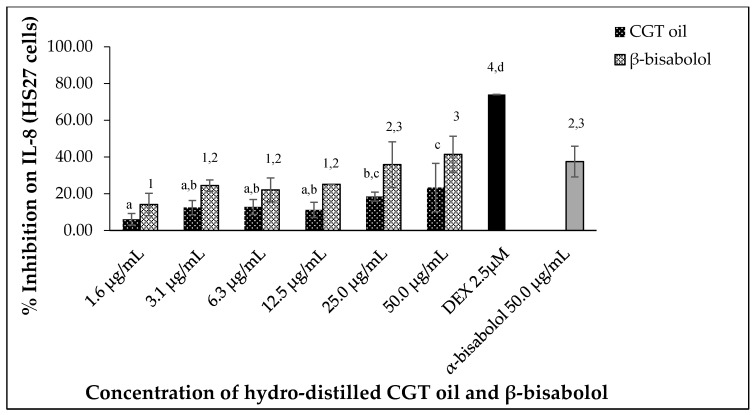
Effect of CGT oil and β-bisabolol dilutions on IL-8 production in HS27 cells. HS27 cells were exposed to test extracts or compounds for 1 h and then induced with 100 ng/mL lipopolysaccharide (LPS) for IL-8 production (*n* = 3). Values are expressed as mean ± SD. Duncan’s multiple range test between average IL-8 production across the different concentrations indicates that dissimilar letters/numerals denote a significant difference (*p* ≤ 0.05).

CGT oil and β-bisabolol downregulated IL-8 production in stimulated human HS27 cells in a dose-dependent manner (Figure 7). Inhibition of IL-8 was 6.1% and 23.4% for the lowest (1.6 µg/mL) and highest (50.0 µg/mL) concentration of CGT oil, respectively. β-Bisabolol at 1.6 µg/mL and 50.0 µg/mL inhibited IL-8 production by 14.2% and 41.5%, respectively. Similar inhibitions were observed for α- and β-bisabolol against IL-8 in HS27 cells at 50.0 µg/mL.

#### 2.5.3. Inhibition of TNF-α

**Figure 8:**
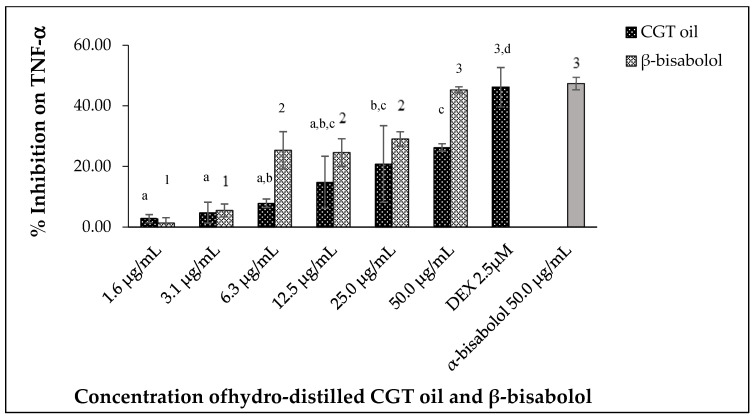
Effect of CGT oil and β-bisabolol dilutions on TNF-α production in RAW 264.7 cells. RAW 264.7 cells were exposed to test extracts or compounds for 1 h and then induced with 100 ng/mL lipopolysaccharide (LPS) for TNF-α production (*n* = 3). Values are expressed as mean ± SD. Duncan’s multiple range test between average TNF-α production across the different concentrations indicates dissimilar letters/numerals denote a significant difference (*p* ≤ 0.05).

Tumor necrosis factor alpha (TNF-α) produced by LPS-stimulated RAW 264.7 macrophages was inhibited by different concentrations of CGT oil and β-bisabolol (Figure 8). CGT oil inhibited TNF-α production by up to 26.3%, whereas β-bisabolol inhibited TNF-α production up to 45.3%. This is close to the level of inhibition observed for the positive control of 2.5 µM (1.0 µg/mL) DEX. There was no significant difference (*p* > 0.05) in inhibitory activity of β-bisabolol (45.3%) and α-bisabolol (47.4%) at 50 µg/mL (Figure 8). The response of macrophage TNF-α production to CGT oil and β-bisabolol exposure was dose-dependent.

### 2.6. Comparing Effect of β-Bisabolol and α-Bisabolol on Two Inflammation Mediators Produced by RAW264.7 Cells

**Figure 9:**
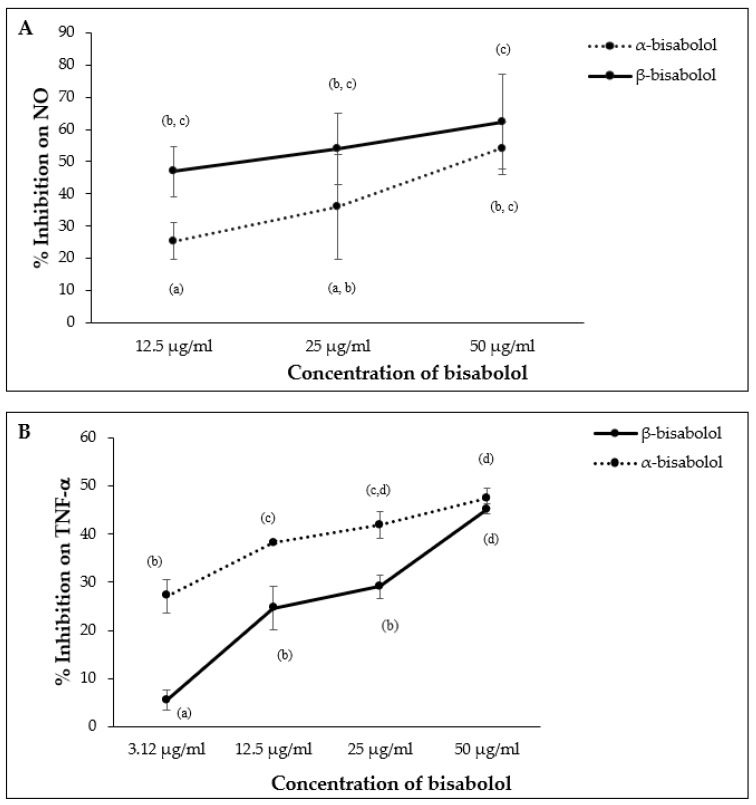
Effect of α-bisabolol and β-bisabolol dilutions on NO (**A**) and TNF-α (**B**) production in RAW 264.7 cells. RAW 264.7 cells were exposed to test extracts or compounds for 1 h and then induced with 100 ng/mL lipopolysaccharide (LPS) for NO and TNF-α production (*n* = 3). Values are expressed as mean ± SD. Duncan’s multiple range test between average TNF-α and NO production across the different concentrations indicates that dissimilar letters/numerals denote a significant difference (*p* ≤ 0.05).

α-Bisabolol and β-bisabolol differed in their effects on NO and TNF-α production in LPS-stimulated macrophages. β-Bisabolol inhibited NO production to a greater extent than α-bisabolol at 12.5 µg/mL, however, the difference between α- and β-bisabolol diminished at 50.0 µg/mL (Figure 9A). In contrast, α-bisabolol inhibited TNF-α production to a greater extent than β-bisabolol at 12.5 µg/mL, but this difference was not apparent at 50.0 µg/mL (Figure 9B). 

## 3. Discussion

The use of essential oils and their chemical components for preventing the overproduction of pro-inflammatory mediators such as NO, PGE_2_, IL-6, and TNF-α has continued to be the focus of research for alleviating symptoms of chronic inflammation and subsequent chronic diseases. In vitro anti-inflammatory assays involve controlling the overproduction of these mediators in stimulated cells. In this study, CGT oil extracted by hydro-distillation and the principal component of the oil, β-bisabolol, were investigated for their anti-inflammatory properties using the 3t3, HS27, and RAW 264.7 cell lines. It was found that a non-toxic concentration range (1.6 µg/mL to 50.0 µg/mL) of CGT oil extracted by hydro-distillation inhibited overproduction of inflammatory mediators by LPS stimulated cells. The anti-inflammatory effect of CGT oil can be attributed to the different bioactive compounds in the oil, which can either work individually or synergistically to cause the inhibition. The process of inhibition of inflammatory mediators involves the regulation of different effectors to the process of inflammation in the body [56]. These effectors include enzymes that can either trigger the production of mediators when cells are stimulated.

Synthesized from L-arginine and molecular oxygen, the signaling molecule nitric oxide is actively involved in innate immunity and inflammation [13,57] and is produced by cells including macrophages actively involved in immune regulation [58]. Regulating nitric oxide production during inflammation is necessary to avoid the progression of acute inflammation to chronic. The regulatory activity of β-bisabolol and CGT oil against NO production by LPS-stimulated macrophages in this experiment indicates that CGT is a possible anti-inflammatory agent for regulating chronic inflammation. Hydro-distilled CGT oil contains some well-known bioactive terpenoids such as β-caryophyllene [59], α-humulene [48], and caryophyllene oxide [60], which regulate NO production during inflammation. The mode of action of essential oils in controlling NO production by macrophages involves inhibiting the enzyme nitric oxide synthase (iNOS), which is responsible for catalyzing NO production in stimulated cells [61,62]. The activity of another key player, nuclear factor kappa-light-chain-enhancer of activated B cells (NF-_K_B), which also stimulates cells during inflammation to produce NO and other pro-inflammatory cytokines, is also regulated in the presence of essential oils [47]. It can therefore be deduced that β-bisabolol and CGT oil inhibition of NO production observed in stimulated RAW 264.7 macrophages could be a result of the inhibition of these key enzymes in the inflammation process. 

Inhibition of PGE_2_ observed in the study also followed a similar pattern to what was observed for NO production in RAW macrophages. Production of PGE_2_ by immune cells contributes to inflammation by promoting the further production of cytokines that are involved in the immune response. Expression of either pro-inflammatory or anti-inflammatory mediators by macrophages depends on the type of stimuli inducing inflammation [32]. Inhibition of PGE_2_ secretion by LPS-stimulated macrophages in the presence of CGT oil were compared with other PGE_2_ assays that report the efficacy of essential oils and terpenoids in reducing inflammation [2,6,48,54]. Similar to NO production in cells, PGE_2_ production during inflammation is triggered by the activity of the enzymes COX-1 and COX-2. The dose dependent control of PGE_2_ production in this study may therefore be linked to the inhibition of COX enzyme activity in the cell. This assumption is supported by other studies that report PGE_2_ and COX inhibition in blood cells and RAW 264.7 macrophages by essential oils from plant materials such as *Chamaecyparis obtusa* [63] and *Trachydium roylei* [2]. The efficacy of β-bisabolol and CGT oil against the overproduction of PGE_2_ was such that at the very low concentration of 1.6 µg/mL, there was an observed inhibition of 35.2% for the oil. Likewise, the high inhibition of PGE_2_ production by β-bisabolol (62.3%, similar to the positive control) at 50 µg/mL further supports the efficacy of β-bisabolol against prolonged inflammation.

The cytokines TNF-α and IL-6, which are well known mediators in the inflammation process, have also been investigated in anti-inflammatory assays when screening oils and compounds for therapeutic properties. Interleukin 6 (IL-6) is one of the cytokines produced by fibroblasts and macrophages that can simultaneously act as a stimulant for the production of other cytokines and acute-phase proteins during inflammation [22,36]. TNF-α is also one of the key players in immune reactions, functioning as a pro-inflammatory cytokine and also as a stimulant for the production of other cytokines and pro-inflammatory mediators such as PGE_2_ [26]. The observed reduction in TNF-α and IL-6 production by CGT oil suggests the ability of β-bisabolol and the oil to control other acute-phase proteins and cytokines, which contribute to prolonging the inflammation process in the body system.

Reduction in cytokine production by CGT oil can also be attributed to the presence of bioactive terpenoids in the oil. This assumption is due to reports from previous anti-inflammatory studies on essential oils from plant materials such as eucalyptus and rosemary inhibiting both IL-6 and TNF-α production in inflamed cells [47]. According to these studies, terpenoids in the oils were the main contributors to IL-6 and TNF-α reduction in cells exposed to the essential oils. One of these studies by Rodrigues et al. [64] suggested that the monoterpenoid eugenol in clove oil was mainly responsible for the reduction in IL-6 in mice macrophages. Likewise, the inhibitory activity of linalool found in aromatic plants is particularly responsible for oils rich in the compound to inhibit TNF-α production in inflammatory cells [65]. A similar regulatory activity was observed for the β-bisabolol in CGT oil against TNF-α and IL-6 production. 

Inhibition of IL-8, a member of the CXC family of chemokine proteins and a chemoattractant [66,67], was also observed for 3t3 fibroblasts exposed to β-bisabolol and CGT oil. The production of IL-8 by the stimulated 3t3 cells, which conforms to previously reported production of the chemokine by fibroblasts [26,68], was also reduced in a dose dependent manner by increasing concentrations of β-bisabolol and CGT oil. IL-8 has a target specificity for neutrophils, activating and directing the migration of neutrophils to the point of infection during inflammation [12,69]. In this manner, IL-8 plays an active role in acute inflammation but prolonged production of the cytokine can lead to chronic inflammation [12]. Therefore, for therapeutic purposes, regulation of IL-8 production is essential for combating inflammatory diseases. Since IL-8 targets neutrophils during inflammation, the regulatory effects of CGT oil on IL-8 production shown in this study suggests that CGT oil could contribute to regulating neutrophil migration during inflammation, thereby inhibiting prolonged inflammation.

The current study suggests β-bisabolol in CGT oil is one of the main contributors to the anti-inflammatory property of the oil. The sesquiterpene alcohol, which made up approximately one-fourth of the total volatile extractives, was effective against all inflammatory mediators investigated. β-Bisabolol has not been investigated for any anti-inflammatory activities prior to this study, even though its isomer α-bisabolol has been extensively studied for anti-inflammatory and other biological activities. Activities associated with α-bisabolol include anti-inflammatory [49,54], anti-oxidant [70], and anti-cancer [71,72]. α-Bisabolol limits the secretion of pro-inflammatory mediators during chronic inflammation [49,54] and in this study, it was demonstrated that β-bisabolol, which differs from α-bisabolol by the position of a hydroxyl group (–OH), also has anti-inflammatory properties.

Comparing the level of β-bisabolol activity with α-bisabolol activity as presented in this study, it appears β-bisabolol was more active against NO production in RAW 264.7 cells than α-bisabolol. One of the properties of α-bisabolol that enhances its anti-inflammatory function is its strong binding affinity to the active sites of pro-inflammatory proteins [49]. The high binding affinity to active sites of pro-inflammatory proteins is associated with compounds of therapeutic properties [73] and this could be the case with β-bisabolol activity in this study, but would need to be tested. 

The reported low EC_50_ values of 1.5 µg/mL (6.8 µM) and 4.3 µg/mL (19.6 µM) for PGE_2_ and IL-6 inhibition, respectively, is indicative that β-bisabolol at low concentrations can be as effective as other anti-inflammatory agents such as resveratrol, dexamethasone, and quercetin [74]. There are reports of α-bisabolol activity on the production of inflammation mediators such as TNF-α, IL-6, IL-1β, and PGE_2_, but limited information on its activity on IL-8 production. The inhibitory effect of α- and β-bisabolol on IL-8 production presented in this study shows that both isomers of bisabolol regulate IL-8 production during prolonged inflammation. Downregulation of inflammatory mediators by β-bisabolol at a level comparable to the similar concentration of α-bisabolol in this study gives a clear indication that β-bisabolol could be used for similar purposes as α-bisabolol. 

Anti-inflammatory activity of β-bisabolol in this study represented by inhibition of cytokines clearly indicates that this sesquiterpene has the property to induce downregulation of the enzymes INOS and COX-2, which are upregulated during inflammation in the body system. Although not investigated in this study, there is a published report on the mechanism of action of α-bisabolol, an isomer of beta-bisabolol [54]. The study reports the downregulation of the enzymes by inhibiting the transfer of signals for their release. α-Bisabolol can inhibit ERK and P-38 signal transmission, induce downregulation of the INOS and COX-2 enzymes, and subsequently reduce NO and PGE2 production in inflamed cells. The downregulation of cytokines observed in our study could follow a similar mechanism.

Although α-bisabolol can be extracted from cultivated chamomile flower, most of it is still sourced from the bark and stems of wild harvested candeia trees due to the high concentration of α-bisabolol [75] and lower cost, although such practice is not sustainable to meet the increasing demand of this bioactive terpenoid. As CGT is readily available as a by-product in large quantity, extracting and exploring β-bisabolol as an alternative topical anti-inflammatory agent to α-bisabolol would be a more cost effective and environmentally friendly approach.

There may be a synergistic anti-inflammatory effect for the major terpenoid compounds in the CGT distilled oil. However, the main purpose of this current study was to find a sustainable source of bioactive bisabolol compounds. The distilled CGT oil will unlikely be used directly as an anti-inflammatory agent as it may have harmful contaminants from the CGT, a by-product of cotton processing. For a large industrial-scale production of bisabolol, the extract from CGT will have to go through a process of cleaning and purification to remove any contaminants, and such a process should ideally also remove compounds other than β-bisabolol. Therefore, the synergistic anti-inflammatory effects of major terpenoid compounds extracted from CGT was not investigated in this study.

## 4. Materials and Methods

### 4.1. Extraction of CGT Oil and Chromatographic Analysis

Essential oil was extracted from pesticide-free CGT, which was comprised mainly of the calyx (bracts). Pesticide-free CGT was kindly supplied by the Australian Cotton Research Institute, Narrabri, NSW, Australia. Milled samples were extracted by means of hydro-distillation, as outlined in Egbuta et al. [50], and extracted oil was collected into a clean 22 mL vial. Extracted CGT oil was stored in the refrigerator at 4 °C for further use. In order to determine the chemical composition of the extracted oil, 50 µL (45.6 mg) CGT oil was diluted in 950 µL (623.9 mg) hexane and analyzed using an Agilent 6890A GC instrument (Wilmington, DE, USA) equipped with a ZB-5 capillary column (Phenomenex) with the dimensions 30 m length × 0.53 mm internal diameter (I.D) × 1.50 µm film thickness. Conditions for the semi-quantitative analysis were the same as the conditions applied by Egbuta et al. [50]. 

### 4.2. Cell Lines and Reagents

Human foreskin fibroblast cells (normal/non-disease, HS27, CRL-1634), mouse embryo fibroblast (3t3, CRL-3242) cells, and murine leukemic macrophages RAW 264.7 (TIB-71) cells were purchased from American Type Culture Collection (ATCC), Manassas, VA, USA. Dulbecco’s modified Eagle medium (DMEM), L-glutamine, sodium pyruvate, phosphate buffered saline (PBS), thiazolyl blue tetrazolium bromide (MTT), dimethyl sulfoxide (DMSO), calf serum, Greiss reagent, penicillin-streptomycin solution, 0.25% trypsin- 0.2 g EDTA, *Escherichia coli* lipopolysaccharide (LPS), dexamethasone (DEX), chlorambucil, and α-bisabolol standard were purchased from Sigma Aldrich Co. Ltd., Burlington, MA, USA.

### 4.3. Cell Culture

Under sterile conditions, Hs27, 3t3, and RAW 264.7 cells were added to 75 cm^2^ Corning^®^ T surface culture flasks (Sigma Aldrich Co. Ltd., Burlington, MA, USA) containing DMEM, 10% calf serum, 2 mM glutamine, 1 mM sodium pyruvate, and penicillin-streptomycin solution. Cells were incubated at 37 °C in a 5% CO_2_ incubator for proper attachment and proliferation. Culture medium was replaced regularly every two days and the cells were checked under a microscope until proliferated up to 90% confluency. When cells achieved approximately 90% confluence, culture medium was removed, and cells washed with 1 mL PBS. Fibroblasts were harvested by trypsinization for the HS27 and 3t3 lines using 3 mL trypsin-EDTA and by scraping for RAW 264.7 cells. Cells were sub-cultured to obtain the required number of cells for subsequent assays. Cell passages used in this study were between passage 2 and passage 8.

### 4.4. Cell Viability Assay

A cell viability assay was performed to determine the non-cytotoxic concentration range ideal for anti-inflammatory assays. Harvested cells were transferred into 96-well plates in triplicate at a seeding density of 1.2 × 10^5^ cells per mL of culture medium (phenol red free) and incubated for 24 h to allow the cells to attach to the bottom of the wells. The seeding density was determined after harvesting the cells and performing cell counting. After 24 h incubation, the cells reached between 85–95% confluency and were then exposed to concentrations of CGT oil between 2.0 µg/mL and 1000.0 µg/mL for a duration of 24 h. Alternatively, cells were exposed to α-bisabolol and β-bisabolol in a range of concentrations between 1.6 µg/mL to 100.0 µg/mL. Positive and negative controls used in the assay were 0.1% DMSO and 1 mg/mL chlorambucil, respectively. Test samples including CGT oil, α-bisabolol, β-bisabolol, and chlorambucil were first dissolved in DMSO and added to culture medium prior to exposure to the cells. An equal concentration of 0.1% DMSO was maintained in CGT oil, α-bisabolol, β-bisabolol, and chlorambucil dilutions. After 24 h exposure to the test compounds, culture media containing test compounds and controls were replaced with 0.3 mg/mL tetrazolium dye (MTT) and incubated for 3 h in the dark at 37 °C. Insoluble formazan crystals formed by viable cells after exposure to MTT for 3 h were solubilized with 200 µL DMSO added to each well and colorimetric absorbance determined at 570 nm with a KC4 multi-detection microplate reader (Bio-Tek Instrument, VT, USA) after 15 min of incubation. Cell viability was calculated using the following equation,

(1) $$\mathrm{Cell}\;\mathrm{viability}=\frac{\mathrm{absorbance}\;\mathrm{of}\;\mathrm{test}/\mathrm{control}}{\mathrm{absorbance}\;\mathrm{of}\;\mathrm{blank}}\times 100$$

where blanks were cells cultured without test compounds or controls. The concentration range used for cell viability assay for α- and β-bisabolol was also selected after the MTT assay was performed.

### 4.5. PGE_2_ Inhibition Assay

Measurement of LPS-induced PGE_2_ production by RAW 264.7 and HS27 cells was conducted using a Cayman PGE_2_ Express ELISA Kit (Cayman Chemical, Ann, Arbor, MI, USA). Cells were seeded at 1.2 × 10^5^ cells per mL of phenol-red free culture medium in 96-well plates and left to incubate overnight for 24 h. After 24 h incubation, the cells were exposed to non-toxic concentrations of CGT oil, β-bisabolol, α-bisabolol, and dexamethasone. Positive (2.5 µM (1.0 µg/mL) DEX) and negative (0.1% DMSO) controls were used and 100 ng/mL LPS was used to stimulate the cells 1 h after adding the test compounds [56]. After a further 24 h incubation, supernatants were collected from each well and PGE_2_ production was analyzed following the manufacturer’s instructions. Measurement of PGE_2_ production was by absorbance at 405 nm with a PerkinElmer VICTOR X4 2030 Multilabel plate reader (PerkinElmer, Seer Green, UK). A standard curve was constructed from dilutions of the PGE_2_ standard and used to determine the concentration of PGE_2_ produced by cells in the assay.

### 4.6. Nitric Oxide Inhibition Assay

Nitric oxide production was measured by quantifying nitrite in the cell supernatant according to previously published procedures [76,77,78] with some modification. RAW 264.7 cells were seeded at a density of 6 × 10^5^ cells per mL of phenol-red free culture medium and incubated for 24 h at 37 °C in a CO_2_ incubator. Cells were incubated with test compounds and controls 1 h before the addition of 100 ng/mL LPS [76]. Dexamethasone (2.5 µM (1.0 µg/mL)) was used as the positive control and 0.1% DMSO as the negative control. Twenty four (24) hours after the addition of LPS, the supernatant was collected and the nitrite concentration analyzed. Greiss reagent was prepared by mixing the purchased Greiss reagent (Sigma Aldrich, Burlington, MA, USA) with 250 mL milli-Q water according to the manufacturer’s instructions. Equal volumes of prepared Greiss reagent and cell supernatant were mixed and incubated for 15 to 20 min in the dark at room temperature before absorbance readings at 550 nm were taken using a PerkinElmer VICTOR X4 2030 Multilabel plate reader (PerkinElmer, Seer Green, UK). A standard curve to quantify nitrite in cell supernatant from absorbance readings was prepared using sodium nitrite standards.

### 4.7. Cytokine Inhibition Assay

A cytokine inhibition assay was conducted to understand the effect of CGT oil and β-bisabolol on LPS-induced production of pro-inflammatory cytokines in HS27, 3t3, and RAW 264.7 cells. Cells were seeded at a density of 1.2 × 10^5^ cells per mL of phenol-red free culture medium and left to incubate for 24 h. The cells were then exposed to non-toxic concentrations of CGT oil, α-bisabolol, and β-bisabolol in triplicate. Controls for the assay were 2.5 µM (1.0 µg/mL) DEX (positive) and 0.1% DMSO (negative). One hour after the addition of test compounds and controls, 100 ng/mL LPS was added to the wells and left to incubate for another 24 h [76]. Cell supernatants were collected and stored at −20 °C following 24 h incubation with LPS. Measurement of cytokines produced by cells exposed to CGT oil and bisabolol isomers was by a BD cytometric bead array (CBA) human and mouse inflammatory cytokines kits (BD Biosciences, East Rutherford, NJ, USA). Following the manufacturer’s instructions, cell supernatants were prepared and analyzed for the production of cytokines using a BD FACSCanto II flow cytometer (BD Biosciences, East Rutherford, NJ, USA) fitted with a 488 nm and 633 nm laser. Parameter voltage values set for the filters were as follows: FSC-290, SSC-200, APC-297, PE-357, FITC-462, SSC threshold fixed at 650 and FSC threshold fixed at 5000. Samples were analyzed alongside prepared cytokine standards between the range of 0 pg/mL to 5000 pg/mL. Data generated from flow cytometry analysis was processed using the FCAP Array Infinite software (Soft flow, V.2.0.0103, Pecs, Hungary). The concentration of cytokines was calculated using a standard curve generated for the different cytokine standards analyzed.

### 4.8. Data Analysis

Data obtained from cell viability, NO, and PGE_2_ assays were analyzed using GraphPad Prism version 4.0 to calculate the concentration of inflammation mediators, IC_50_ and EC_50_ of CGT oil and β-bisabolol. Microsoft Excel 2013 was used to calculate the mean concentration and standard deviation of the different inflammatory mediators measured. Analysis of variance (ANOVA) was performed using GenStat 64-bit Release 18.1 (18th edition) with the Duncan multiple range test. *p* values ≤ 0.05 were considered to indicate statistical significance.

## 5. Conclusions

CGT oil and β-bisabolol isolated from CGT oil inhibited the production of NO, PGE_2_, TNF-α, IL-6, and IL-8 in RAW 264.7, 3t3, and HS27 cells in a dose-dependent manner. β-Bisabolol was the most abundant terpenoid in CGT oil and the primary contributor to the anti-inflammatory activity of the oil. The sesquiterpenoid, β-bisabolol had similar anti-inflammatory effects as its isomer α-bisabolol and in some cases was more effective than α-bisabolol as observed for NO and IL-6 inhibition. Compared to the composition reported for α-bisabolol (7.7–16.4%) in chamomile flower extracts, the higher concentration of β-bisabolol in CGT oil (23.5%) and its high anti-inflammatory activity first reported in this study suggests that this terpenoid could be used as an alternative to its isomer α-bisabolol for the treatment of inflammatory conditions. The observed activity of β-bisabolol provides an indication of its potential anti-inflammatory activity when investigated in an in vivo setting against the well-established bioactive α-bisabolol. Although not investigated in the present study, an in vivo anti-inflammatory assay of β-bisabolol alongside α-bisabolol will further validate the efficacy of β-bisabolol as an anti-inflammatory agent. This assay can be performed in the future following ethical considerations and strict adherence to EU limitations on in vivo assays using cosmetic products.

## Figures and Tables

**Table 1:** Terpenoid composition of pesticide-free CGT oil extracted by hydro-distillation.

Terpenoids	% Composition
β-bisabolol	23.5
γ-bisabolene	8.7
β-caryophyllene	6.8
caryophyllene oxide	4.7
α-humulene	4.1
α-cuprenene	4.1
β -ocimene	3.9
gossonorol	2.8
β-santalene	2.0
α-pinene	1.3
α-copaene	1.2
myrcene	1.1
sesquisabinene	1.1
(*E*)-nerolidol	0.8
italicene	0.7
β-bisabolene	0.6
5-hydroxy-cis-calamenene	0.5
β-pinene	0.4
epi-β-santalene	0.3
α-santalene	0.3
γ-curcumene	0.3
β-copaene	0.2
α-curcumene	0.2
γ-terpineol	0.02

## Data Availability

All available data has been published with this article.

## Supplementary Materials

The following are available online. Table S1: Percentage abundance of volatiles identified in hydro-distilled pesticide-free CGT extracts.

## Sample Availability

Samples of the compounds are not available from the authors.
