# Supplementary material for: In Vitro Anti-Inflammatory Activity of Essential Oil and β-Bisabolol Derived from Cotton Gin Trash
Source: Molecules. 2022 Jan 14;27(2):526. doi: 10.3390/molecules27020526 (PMC8779114; doi:10.3390/molecules27020526)
Supplement: Supplementary file 1 [file molecules-27-00526-s001.zip › molecules-1481767-supplementary.pdf]

Article

In Vitro Anti-Inflammatory Activity of Essential Oil and β-Bisabolol Derived from Cotton Gin Trash

Mary A. Egbuta [1], Shane McIntosh [1], Daniel L. E. Waters [1], Tony Vancov [2] and Lei Liu [1,*]

[1] Southern Cross Plant Science, Faculty of Science and Engineering, Southern Cross University, Lismore, NSW 2480, Australia; Mary.Egbuta@scu.edu.au (M.A.E.); Shane.McIntosh@scu.edu.au (S.M.); daniel.waters@scu.edu.au (D.L.E.W.)

[2] Elizabeth Macarthur Agricultural Institute, NSW Department of Planning, Industry & Environment, DPI Agriculture, Woodbridge Rd, Menangle, NSW 2568, Australia; tony.vancov@dpi.nsw.gov.au

* Correspondence: ben.liu@scu.edu.au; Tel.:+61-02-6620-3293

Supplementary Table S1. Percentage abundance of volatiles identified in hydro-distilled pesticide-free CGT extracts.

**Volatile compounds**	**% abundance**
α-pinene	1.27
myrcene	1.13
β-ocimene	3.91
N-methylpyrrole	0.33
γ-terpineol	0.02
α-copaene	1.16
α-cuprenene	4.08
α-santalene	0.32
caryophyllene(E-)	6.81
epi-β-santalene	0.33
β-santalene	1.95
α-umulene	4.08
γ-curcumene	0.29
ar-curcumene	0.15
β-copaene	0.15
sesquisabinene	1.09
3-ethyl-4-methyl-5-(methylthio)isothiaz	0.23
β-bisabolene	0.60
1-ethyl-2,3-dimethyl-benzene	7.26
(Z)-γ-Bisabolene	8.68
1H-Cyclopenta[1 ,3]-β-cubebene	0.92
2,4-dimethyl-quinoline	1.76
bicyclo[4.1.0]hept-3-carene	7.94
2,3,6,7,8,8a-he-1H-3a,7-methanoazulene	1.68
dodecanoic acid	0.23
2,6-bis(1,1-dimethylethyl)-4-me-phenol	0.18
1,2,4,5-tetramethyl-benzene	0.63
nerolidol	0.75

3,4,4-trimethyl-2-cyclohexen-1-one	0.29
2-ethyl-1,4-dimethyl-benzene	0.13
vulgarol B	0.31
1-(2-aminophenyl)-1-(4-pyridyl)ethane	0.29
1-naphthalenyl-thiourea	0.14
caryophyllenyl alcohol	0.23
1-methylene-3-cyclohexane	0.68
2-Hydroxyfluoranthene	0.21
caryophyllene oxide	4.65
2,3-dimethyl-thiophene	0.07
α-himachalene	0.23
trans-3,4-dimethyl-1-cyclohexenecarbald	0.25
gossonorol	3.24
1-(hydroxymethyl)-2-vinyl-2-isopropylcy	4.10
4-cyclohexyl-benzenamine	0.18
β-bisabolol	23.50
isocamphane	0.43
italicene	0.68
1,2,4,5-tetramethyl-benzene,	0.12
1,3,5-bisabolatrien-7-ol	0.09
tetradecanoic acid	0.12
4-oxotricyclo[3.3.1.13]-thiocyanic acid	0.17
cadina-4,10(15)-dien-3-one	0.04
5-hydroxy-cis-calamenene	0.54
6,10,14-trimethyl-2-pentadecanone	0.17
2H-isoindole	0.11
n-pentadecanol	0.10
9-bromo-9-methyl-bicyclo[6.1.0]nonane	0.02
5-methyl- 1,3,6-heptatriene	0.02
5Z,9E-farnesyl acetone	0.09
1-nonadecene	0.20
n-hexadecanoic acid	0.29
2Z,6Z-farnesol	0.39
